# Dexmedetomidine is safe and effective for reducing intraprocedural pain in colorectal endoscopic submucosal dissection

**DOI:** 10.1002/deo2.223

**Published:** 2023-03-27

**Authors:** Hiroyoshi Iwagami, Takuji Akamatsu, Kazuki Matsuyama, Yusuke Hanawa, Kohei Tonomura, Eiki Chikugo, Shinya Ogino, Hiroki Morimura, Masayuki Shimoyama, Tomoko Terashita, Shogo Nakano, Midori Wakita, Takeya Edagawa, Takafumi Konishi, Hisakazu Matsumoto, Yasuki Nakatani, Shunji Urai, Takeshi Seta, Yoshito Uenoyama, Yukitaka Yamashita

**Affiliations:** ^1^ Department of Gastroenterology and Hepatology Japanese Red Cross Wakayama Medical Center Wakayama Japan

**Keywords:** dexmedetomidine, endoscopic submucosal dissection, colorectal, anesthesia, sedation

## Abstract

**Objectives:**

Endoscopic submucosal dissection (ESD) is effective for the resection of colorectal intramucosal lesions. This study was performed to examine the safety and effectiveness of using dexmedetomidine (DEX) in the anesthesia regimen of patients with colorectal lesions undergoing ESD.

**Methods:**

We retrospectively examined 287 consecutive patients who underwent ESD for colorectal lesions in our institution from January 2015 to December 2021. Outcomes including the frequency of intraprocedural pain and adverse events were compared between the DEX and no DEX groups. Moreover, univariate and multivariate analyses were conducted for each clinical factor of intraprocedural pain. Intraprocedural pain was defined as patient‐reported abdominal pain or body movement during the procedure.

**Results:**

The incidence of intraprocedural pain was significantly lower in the DEX than in the no DEX group (7% vs. 17%, *p* = 0.02). The incidence of hypotension was also significantly higher in the DEX group (7% vs. 0%, *p* = 0.01), but no cerebrovascular or cardiac ischemic events occurred. In the univariate analyses, the diameter of the resected specimen, procedure time, no use of DEX, and total midazolam dose was associated with intraprocedural pain. The midazolam dose and DEX administration were significantly negatively correlated and the diameter of resected specimen and procedure time were significantly positively correlated. Multivariate logistic regression showed that no use of DEX was independently associated with intraprocedural pain (*p* = 0.02).

**Conclusions:**

Adding DEX to the anesthesia regimen in patients undergoing colorectal ESD appears to be safe and effective for reducing intraprocedural pain.

## INTRODUCTION

Colorectal cancer is the fourth most common cancer worldwide.^1^ Early detection and treatment are important to reduce colorectal cancer mortality.^2^ Endoscopic resection is mainly performed for intramucosal lesions. Endoscopic resection techniques include cold snare polypectomy, hot snare polypectomy, endoscopic mucosal resection (EMR), and endoscopic submucosal dissection (ESD). En‐bloc resection is recommended because piecemeal resection is a risk factor for recurrence.^3^, ^4^, ^5^, ^6^ According to the guidelines of the European Society of Gastrointestinal Endoscopy,^7^ American Gastroenterological Association,^8^ and Japanese Society of Gastroenterology,^9^ EMR or ESD is recommended for lesions ≥20 mm in size and those comprising ≥50% of the circumference of the colorectal lumen. Because ESD is associated with a higher en‐bloc resection rate than EMR for lesions ≥20 mm,^10^ ESD remains important in the surgical treatment of intramucosal colorectal cancer. In many countries, ESD is performed under intravenous anesthesia in an endoscopy room rather than under general anesthesia in an operating room for various reasons such as anesthesiologist availability. In such cases, patient safety and adequate pain control during the procedure are important.

The 2020 Japan Gastroenterological Endoscopy Society guidelines for sedation in endoscopy noted the usefulness of dexmedetomidine (DEX) in the sedation of patients undergoing transanal endoscopic resection.^11^ DEX is reportedly effective for suppressing patient body movements in long‐duration procedures such as gastric ESD and endoscopic retrograde cholangiopancreatography.^12^, ^13^ In a randomized controlled trial of DEX for conscious sedation in colorectal ESD,^14^ both patient and endoscopist satisfaction were superior in the pethidine (an analgesic agent) and DEX group than in the pethidine and placebo group. However, in real‐world clinical practice, anesthesia for colorectal ESD is often administered using a combination of analgesic and sedative agents rather than an analgesic agent alone. To better reflect real‐world practice, this study was performed to compare safety and outcomes between anesthesia with combined analgesic and sedative agents and anesthesia with these combined agents and DEX.

## PATIENTS AND METHODS

### Patients

This retrospective study was conducted at the Japanese Red Cross Wakayama Medical Center, a tertiary general hospital in Wakayama, Japan. Consecutive patients in our endoscopic and pathological database who underwent ESD for colorectal lesions from January 2015 to December 2021 were eligible for enrollment. Those whose procedure was interrupted because of technical factors such as severe fibrosis were excluded. The study was performed in accordance with the principles of the Declaration of Helsinki. Institutional review board approval was obtained.

### Evaluated items and definitions of outcomes and adverse events

Outcomes including the frequency of intraprocedural pain and adverse events were compared between the DEX and no DEX groups. Moreover, univariate and multivariate analyses were conducted for each clinical factor of intraprocedural pain. Several factors other than DEX appear to be related to intraprocedural pain in daily practice. We investigated the factors related to intraprocedural pain to confirm whether DEX was independently related to no intraprocedural pain. Based on our experience in daily practice, we considered that the presence of dementia or the patient's performance status might be associated with physical movement and that the patient's surgical history or the endoscopist's experience might be associated with abdominal pain. We therefore analyzed several factors associated with intraprocedural pain including dementia, a history of an abdominal operation, and others.

The definition of intraprocedural pain during ESD was not judged by the patient's distressed expression but by the patient's direct complaint of pain. Physical movements of patients that required temporary interruption of ESD were included in the definition of intraprocedural pain. We retrospectively searched the patients’ data in their intraoperative examination records and nursing records. Patients who had at least one event of intraprocedural pain or physical activity were categorized as the pain group and those who did not were categorized as the no‐pain group. Procedure time was defined as the time from injection of normal saline or purified sodium hyaluronate to ESD completion. Intraprocedural bleeding was defined as the spurting of blood or persistent oozing that did not spontaneously arrest within 60 s or following water irrigation, requiring endoscopic hemostasis with endoclips or coagulation. Intraprocedural perforation was defined as an iatrogenic defect in the muscle layer confirmed by visualization of fatty tissue or other organs through the defect. Delayed bleeding was defined as postprocedural bleeding that required presentation to the emergency department, hospitalization, or medical intervention. Delayed perforation was defined as postprocedural perforation diagnosed by the presence of free air or fluid on abdominal computed tomography or plain radiography in a patient who had no evidence of perforation immediately after the procedure. We defined an “expert” as an endoscopist with Japanese endoscopy specialist certification and more than 10 years of experience. Other endoscopists were defined as “non‐experts.”

### Diagnosis of colorectal lesions

Endoscopic resection was performed for adenomas, sessile serrated lesions, large hyperplastic polyps, intramucosal carcinoma (high‐grade adenoma/dysplasia according to the Vienna classification^15^), and superficial submucosal invasive carcinoma. In accordance with European and Japanese guidelines,^7^, ^16^ we generally perform ESD for large lesions with a diameter of ≥50% the circumference of the lesion and for suspected superficial submucosal invasive carcinoma. By contrast, lesions with an expansive appearance, erosion/ulceration, fold convergence, stiffness, and elevation in a depressed area^5^, ^17^ and those classified as Japan NBI Expert Team type 3^18^ were diagnosed as deeply submucosal invasive carcinoma. Patients with such lesions underwent surgical resection. The lesion size was estimated based on its endoscopic appearance.

### Anesthesia administration

Midazolam and pethidine hydrochloride were used in all patients. DEX was additionally used in patients who underwent ESD after 2018. Patients who received midazolam and pethidine hydrochloride were categorized as the no DEX group; those who received midazolam, pethidine hydrochloride, and DEX were categorized as the DEX group. In the no DEX group, the loading doses of midazolam and pethidine hydrochloride were 0.02–0.03 mg/kg and 17.5–35 mg, respectively. In the DEX group, the corresponding doses were 0.01–0.02 mg/kg and 17.5–35 mg, respectively. DEX was administered using a 2 μg/kg/h infusion for 6 min as a loading dose followed by a 0.2 μg/kg/h continuous infusion. In older patients or those with light body weight, the initial dose of pethidine hydrochloride is 17.5mg. If such patients complained of abdominal pain, pethidine hydrochloride was added in 17.5 mg increments. Additional midazolam or DEX was administered as appropriate during the procedure to maintain the Ramsay sedation score at 2–4.^19^ Etilefrine hydrochloride was administered and the dose of DEX was decreased if the systolic blood pressure decreased to <80 mmHg. If the heart rate decreased to <40 beats per minute, atropine sulfate hydrate was administered and the dose of DEX was decreased. If the blood pressure and heart rate still did not improve, DEX was discontinued.

### ESD procedure and pathological evaluation

Procedures were performed using the PCF‐H290ZI or PCF‐H290TI endoscopic system in conjunction with the Evis Lucera Elite CV‐290/CLV‐290 video system (Olympus Co., Tokyo, Japan). Slit and hole caps (Top Co., Tokyo, Japan) of the appropriate size for the endoscope were used. All procedures were performed by a combination of expert and non‐expert endoscopists.

Resected specimens were embedded in paraffin and stained with hematoxylin and eosin. Diagnoses were made by dedicated pathologists according to the Japanese Classification of Colorectal Carcinoma.^20^ Lymphovascular permeation was assessed for cancer lesions using hematoxylin and eosin staining and immunochemical staining. Patients were routinely discharged on day 5 or 6 after the procedure.

### Statistical analysis

Statistical analyses were performed using EZR (Saitama Medical Center, Jichi Medical University, Saitama, Japan)^21^ and SPSS software version 22 (IBM Corp., Armonk, NY, USA). Categorical data are expressed as numbers with percentages and were compared using Fisher's exact test. Continuous data are expressed as median with range and were compared using the Mann–Whitney U test. Correlations were assessed using Pearson's method; an absolute correlation coefficient value >0.4 indicated a correlation. The association of factors with intraprocedural pain was assessed using univariate and multivariate logistic regression; variables significant in the univariate analysis, those that seemed important based on daily practice, and those reported to be significant in a previous study^14^ were used in the multivariate analysis. A *p‐*value of <0.05 was considered statistically significant.

## RESULTS

### Patient and lesion characteristics

**FIGURE 1:**
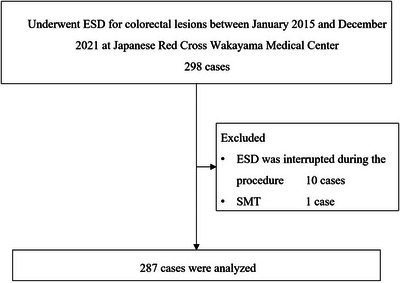
Study flow chart. ESD, endoscopic submucosal dissection; SMT, submucosal tumor.

The study flow chart is shown in Figure 1. Among the 298 eligible patients who underwent ESD for colorectal lesions during the study period, 10 patients with procedural interruption and one patient with a submucosal tumor were excluded. Therefore, 287 patients were included in the analysis. The patient and lesion characteristics according to the anesthetic group are shown in Table 1. The diameter of the resected specimen was significantly larger in the DEX group than in the no DEX group (38 vs. 34 mm, *p* = 0.01). Sex, hypertension, lesion location, and endoscopist experience (expert or non‐expert) also significantly differed between the groups.

**TABLE 1:** Patient and lesion characteristics according to anesthetic group.

		DEX group *n* = 155	No DEX group *n* = 132	*p*‐Value
Age, median (range)		71 (42–94)	69 (37–88)	0.10
Sex	Male	70 (45)	80 (61)	0.01
	Female	85 (55)	52 (39)	
Cardiovascular disease		6 (4)	13 (10)	0.06
Pulmonary disease		3 (2)	4 (3)	0.71
Hypertension		2 (1)	17 (13)	<0.001
Macroscopic type	0‐I	99 (64)	73 (55)	0.40
	0‐IIa	55 (35)	58 (44)	
	0‐IIc	1 (1)	1 (1)	
Tumor location	Right / left colon	99 (64)	107 (81)	0.002
	Rectum	56 (36)	25 (19)	
Endoscopists’ experience	Expert	89 (57)	99 (75)	0.001
	Non‐expert	66 (43)	33 (25)	

Values shown are numbers (percentages) unless otherwise indicated.

Abbreviation: DEX, dexmedetomidine.

### Procedural outcomes and adverse events

Table 2 shows the procedural outcomes and adverse events according to the anesthetic group. The total midazolam dose (1.0 vs 1.5 mg, *p* < 0.001), the incidence of intraprocedural pain (7% vs. 17%, *p* = 0.02), and intraprocedural perforation rate (0% vs. 5%, *p* = 0.003) were significantly lower in the DEX group. The rates of intraprocedural bleeding and postprocedural fever, perforation, and bleeding did not significantly differ between the groups. The incidence of hypotension was significantly higher in the DEX group (7% vs. 0%, *p* = 0.002). No cerebrovascular events occurred.

**TABLE 2:** Procedural outcomes and adverse events according to the anesthetic group.

		DEX group *n* = 155	No DEX group *n* = 132	*p*‐Value
**Procedure outcomes**				
Total amount of midazolam (mg/kg), median (range)		0.015 (0–0.127)	0.026 (0.009–0.132)	<0.001
Procedure time (minute), median (range)		92 (25–330)	90 (16–420)	0.61
Intraprocedural pain	Presence	11 (7)	22 (17)	0.02
	Absence	144 (93)	110 (83)	
*En bloc* resection	*En bloc* resection	151 (97)	126 (96)	0.52
	Piecemeal resection	4 (3)	6 (4)	
Diameter of resected specimen (mm), median (range)		38 (20–100)	35 (15–90)	0.01
Pathological type	Adenoma	65 (42)	56 (42)	0.30
	SSL	10 (7)	16 (12)	
	Tis	61 (39)	41 (31)	
	T1a	9 (6)	13 (10)	
	T1b	8 (5)	5 (4)	
	Others	2 (1)	1 (1)	
**Complications**				
Intraprocedural perforation		0	7 (5)	0.003
Intraprocedural bleeding		0	1 (1)	0.46
Postprocedural fever up		9 (6)	4 (3)	0.39
Postprocedural perforation		0	0	–
Postprocedural bleeding		7 (5)	2 (2)	0.19
**Side effects**				
Hypoxia^a^		17 (11)	8 (6)	0.21
Hypotension^b^		10 (7)	0	0.002
Bradycardia^c^		4 (3)	0	0.13

Values shown are numbers (percentages) unless otherwise indicated.

Abbreviations: DEX, dexmedetomidine; SSL, sessile serrated lesion.

^a^ Blood oxygen saturation <85%

^b^ Systolic blood pressure <70 mm Hg.

^c^ Heart rate <40 beats per minute.

### Clinical factors associated with intraprocedural pain

Table 3 shows the clinical factors associated with intraprocedural pain in the univariate and multivariate analyses. In the univariate analysis, age, sex, dementia, performance status, history of an abdominal operation, lesion location, and macroscopic type showed no association with intraprocedural pain. Conversely, the diameter of the resected specimen, midazolam dose, no DEX administration, and procedure time were associated with intraprocedural pain. The midazolam dose and DEX administration were significantly negatively correlated (Pearson's correlation coefficient, ‐0.485), and the diameter of the resected specimen and procedure time were significantly positively correlated (Pearson's correlation coefficient, 0.564). In the multivariate analysis, no DEX administration and a long procedure time were independently associated with intraprocedural pain (*p* = 0.02 and *p* < 0.001, respectively).

**TABLE 3:** Clinical factors associated with intraprocedural pain by univariate and multivariate analyses.

		Univariate analysis		Multivariate analysis***	
		Odds ratio (95% CI)	*p*‐Value	Odds ratio (95% CI)	*p*‐Value
Age		−	0.25	−	−
Sex	Male/female (reference)	0.73 (0.33–1.62)	0.46	−	−
Dementia	Positive/negative (reference)	2.6 (0.05–33.53)	0.39	−	−
PS	0/1/2	−	0.28	−	−
Abdominal operation history	Positive/negative (reference)	0.62 (0.07–2.70)	0.75	−	−
Tumor location	Right or left/rectum (reference)	0.89 (0.39–2.21)	0.84	−	−
Diameter of resected specimen*		−	0.02	−	−
Macroscopic type	0‐I/0‐IIa/0‐IIc	−	0.74	−	−
Amount of midazolam**		−	< 0.001	−	−
Dexmedetomidine**	With/without (reference)	0.38 (0.16–0.87)	0.02	0.39 (0.17–0.87)	0.02
Endoscopists’ experience	Expert/non‐expert (reference)	1.47 (0.62–3.70)	0.44	2.00 (0.79–5.00)	0.14
Procedure time*		−	0.006	1.01 (1.01–1.02)	< 0.001

Abbreviations: CI, confidence interval; PS, performance status.

* Diameter of resected specimen and procedure time were significantly positively correlated (Pearson's correlation coefficient, 0.564).

** Midazolam dose and dexmedetomidine administration were significantly negatively correlated (Pearson's correlation coefficient, ‐0.485).

*** Multivariate analysis was performed by three items (dexmedetomidine, endoscopists’ experience, and procedure time); midazolam and the diameter of the resected specimen were excluded.

## DISCUSSION

To the best of our knowledge, this is the first study to investigate the effect of adding DEX to the intravenous anesthesia regimen in patients undergoing colorectal ESD. Our findings suggest that such use of DEX reduces intraprocedural pain.

Although deep sedation is typically required for esophageal or gastric ESD because of procedure‐related pharyngeal and oral discomfort and the procedure's long duration, colorectal ESD requires light or conscious sedation because it requires the patient to change their posture and breathing during the procedure.^14^ Changing posture on command may be required to apply gravity‐assisted traction,^22^ improve endoscopic visualization, and allow certain surgical maneuvers. Moreover, the incidence of colorectal endoscopy‐related adverse events such as hypoxemia is lower with conscious sedation than with deep sedation.

DEX is a potent and highly selective α2‐adrenergic receptor agonist that has sedative and analgesic effects that reduce the required dose of other anesthetic agents.^23^, ^24^ It is commonly used for sedation in the intensive care unit because of its safety and efficacy.^25^ For endoscopic procedures, DEX can be used to maintain adequate conscious sedation or light sedation that allows easy awakening with a verbal or physical stimulus.^14^ Our intraprocedural pain findings demonstrated the analgesic effects of DEX; especially, the incidence of patient‐reported abdominal pain and patient movement during the procedure was lower in the DEX group than in the no DEX group. Moreover, DEX has an immunomodulatory effect that suppresses cytokine production and leads to decreased systemic inflammation.^26^, ^27^, ^28^ DEX may also suppress ESD‐related inflammation.^14^ In our study, the incidence of postprocedural fever did not significantly differ between the DEX and no DEX groups. Although DEX may contribute to a reduced incidence of post‐ESD coagulation syndrome,^29^ further investigation is required.

We believe that the use of DEX in anesthesia for colorectal ESD has several merits. Even when anesthesia with DEX was titrated to maintain a Ramsay sedation score of 2 or 3, patients rarely complained of abdominal pain. They were also able to respond quickly to commands, even when they were asleep. Our univariate analyses showed that a small diameter of the resected specimen, short procedure time, use of DEX, and low total midazolam dose were associated with no intraprocedural pain. In the multivariate analysis, the use of DEX was an independent factor associated with no intraprocedural pain.

However, DEX may have disadvantages as well. The use of anesthetic agents reportedly increases the risk of perforation during colorectal endoscopic resection procedures.^30^ In addition, DEX has been associated with adverse effects such as hypotension, bradycardia, and hypoxemia.^14^ In our study, although the incidence of intraprocedural perforation was lower in the DEX group than in the no DEX group, the incidence rates of intraprocedural bleeding and postprocedural bleeding and perforation did not significantly differ between the two groups. The use of DEX did not increase the incidence of procedural complications. The fact that the incidence of intraprocedural perforation was lower in the DEX group might be attributed to greater endoscopist experience (use of DEX began later in the study period). However, stable sedation by DEX may have also reduced the incidence of perforation. A randomized controlled study in which the sedation level is investigated in detail is required to elucidate the relationship between the sedation level and perforation. With regard to cardiovascular adverse effects, the incidence of hypotension was higher in the DEX group, whereas the incidence rates of bradycardia and hypoxemia did not significantly differ between the groups. All cardiovascular adverse effects rapidly improved with a single dose of a vasopressor agent or atropine. Moreover, no cardiac ischemia or cerebrovascular events occurred. Conscious or light sedation with DEX appears to be safe and effective in patients undergoing colorectal ESD. However, the incidence of pre‐existing cardiac and pulmonary disease was low in our patient cohort.

Our study has several limitations. It was retrospective in design and was conducted in a single center. In addition, our use of patient‐reported pain and body movement during the procedure as an outcome measure was not truly objective. Furthermore, we did not assess patient or endoscopist/nurse satisfaction. However, our findings are promising and warrant confirmation in future prospective randomized studies.

In conclusion, adding DEX to the anesthesia regimen in patients undergoing colorectal ESD appears to be safe and effective for reducing intraprocedural pain.

## CONFLICT OF INTEREST STATEMENT

The authors declare no conflicts of interest.
